# Differential Regulatory Analysis Based on Coexpression Network in Cancer Research

**DOI:** 10.1155/2016/4241293

**Published:** 2016-08-11

**Authors:** Junyi Li, Yi-Xue Li, Yuan-Yuan Li

**Affiliations:** ^1^Key Lab of Computational Biology, CAS-MPG Partner Institute for Computational Biology, Shanghai Institutes for Biological Sciences, Chinese Academy of Sciences, Shanghai 200031, China; ^2^Shanghai Center for Bioinformation Technology, 1278 Keyuan Road, Shanghai 201203, China; ^3^Shanghai Industrial Technology Institute, 1278 Keyuan Road, Shanghai 201203, China; ^4^Shanghai Engineering Research Center of Pharmaceutical Translation, 1278 Keyuan Road, Shanghai 201203, China

## Abstract

With rapid development of high-throughput techniques and accumulation of big transcriptomic data, plenty of computational methods and algorithms such as differential analysis and network analysis have been proposed to explore genome-wide gene expression characteristics. These efforts are aiming to transform underlying genomic information into valuable knowledges in biological and medical research fields. Recently, tremendous integrative research methods are dedicated to interpret the development and progress of neoplastic diseases, whereas differential regulatory analysis (DRA) based on gene coexpression network (GCN) increasingly plays a robust complement to regular differential expression analysis in revealing regulatory functions of cancer related genes such as evading growth suppressors and resisting cell death. Differential regulatory analysis based on GCN is prospective and shows its essential role in discovering the system properties of carcinogenesis features. Here we briefly review the paradigm of differential regulatory analysis based on GCN. We also focus on the applications of differential regulatory analysis based on GCN in cancer research and point out that DRA is necessary and extraordinary to reveal underlying molecular mechanism in large-scale carcinogenesis studies.

## 1. Introduction

In the past decade, plenty of computational methods and algorithms such as differential analysis and network analysis [1, 2] are proposed to explore genome-wide gene expression characteristics with rapid development of high-throughput technologies and accumulation of big transcriptomic data. These efforts in computational genomic area are dedicated to transform underlying genomic information into valuable knowledges in biological and medical research fields [3, 4]. Recently, tremendous integrative research aims to interpret the development and progress of cancers because elucidating molecular regulatory mechanisms, especially the dysregulation mechanisms, of neoplastic diseases makes great benefit in medical and pharmaceutical aspects. Although partial different regulatory functions of cancer hallmarks such as evading growth suppressors and resisting cell death [5] have been revealed, the whole dysregulation mechanisms are far from clear.

Cancer is a complex disease and an effective way to study regulatory role of genes involved in cancer is to summarize them into network [6]. It is suggested that genes having similar or correlated expression patterns might contribute to the same regulatory function and gene coexpression patterns revealed by coexpression network analysis may lead to more insightful discovery on the underlying regulatory mechanisms [2, 7]. By comparing the difference of the regulatory networks between cancer and normal status, specific differential network of genes can be identified as dysfunctional in cancer. A large number of reverse engineering approaches have been developed to construct regulatory network from gene expression data. For examples, Xiao suggested Boolean model to analyze and stimulate the gene regulatory network [8]. Some methods based on Bayesian model lead to Bayesian networks and they are widely applied [9–11]. Nonlinear differential equation model is also developed to construct the regulatory network [12]. Prior biological knowledge such as transcription factor- (TF-) target regulatory relationships or miRNA-target regulatory relationships can also be integrated into modelling framework [11, 13, 14]. These reverse and forward integrated approaches are supposed to have smaller false positive rate to extract informative insights of transcriptomic behaviors.

Although network analysis provides the possibility to comprehensively understand biological processes, it does increase the computational complexity. Decreasing the searching space before network analysis is necessary in high dimension data analysis. An obvious strategy of reducing the computational burden is to build a subnetwork around a given set of genes such as previously reported disease-related genes [15] or around differentially expressed genes [16–18]. Differential expression analysis (DEA) compares the mean expression value of genes between case and control samples and identifies significantly differentially expressed genes by statistical tests. In current transcriptomic analysis procedure, DEA has become the basic and the very first analysis step.

Recently, differential coexpression analysis (DCEA) increasingly plays a robust complement to DEA [2] and is widely used in discovering the system properties of carcinogenesis features. By calculating the change of correlations between gene pairs instead of mean expression level, DCEA provides more information about phenotypic change-related regulatory network [19–24]. Therefore, differential regulatory analysis based on coexpression network may detect more insights into regulatory mechanisms.

In this review, we will introduce the paradigm of differential regulatory analysis (DRA) based on gene coexpression network (GCN). We also focus on the applications of DRA based on GCN in cancer research and point out that DRA is necessary and extraordinary to reveal underlying regulatory mechanism in large-scale carcinogenesis studies.

## 2. Paradigm of Differential Regulatory Analysis Based on Gene Coexpression Network

**Figure 1:**
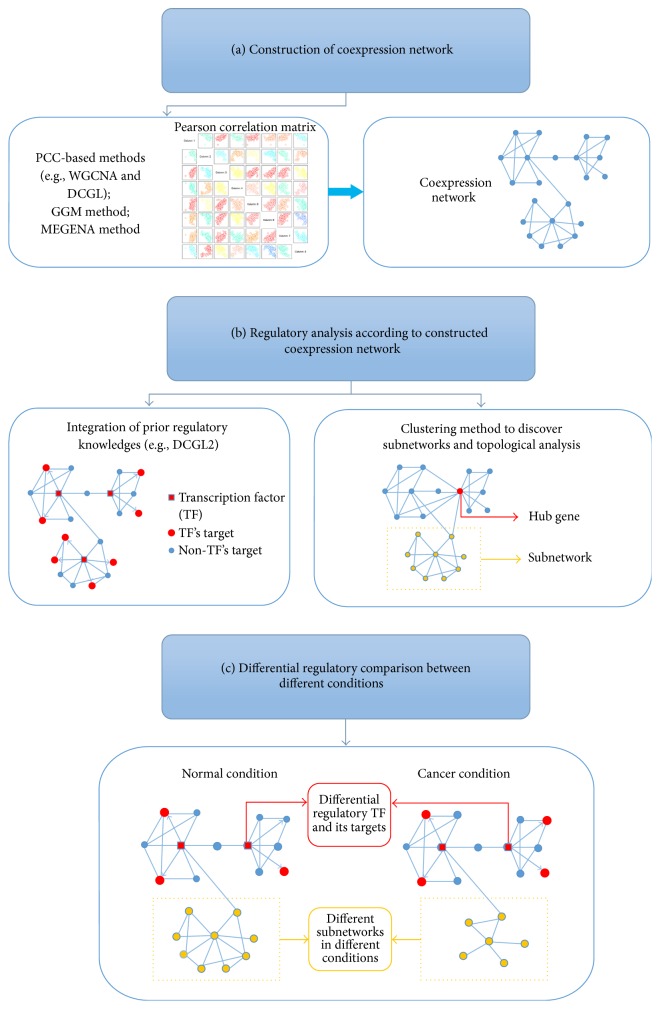
Paradigm of differential regulatory analysis based on gene coexpression network. The paradigm of differential regulatory analysis based on gene coexpression network includes but is not limited to three procedures. (a) Constructing gene coexpression network based on genomic transcriptomic data. (b) Regulatory analysis according to gene coexpression network. (c) Differential regulatory comparison between different conditions.

Differential regulatory analysis based on gene coexpression network has been widely used in carcinogenesis regulation research and basically includes three procedures as shown in Figure 1: constructing gene coexpression network based on transcriptomic data, regulatory analysis according to gene coexpression network, and differential regulatory comparison between different conditions.

### 2.1. Construction of Gene Coexpression Network

In a gene coexpression network, genes are nodes and their correlations are represented by the edges of network. Pearson correlation coefficient (PCC) is the mostly used score to measure the tendency of gene expression correlation [25–28]. The value of PCC ranges from −1 to 1 and higher absolute value of PCC means higher correlation between gene pairs. When constructing gene coexpression network, a correlation threshold is selected. After removing the nonsignificant edges or negligible coordinated gene pairs by the threshold, gene coexpression network is constructed by the significantly correlated gene pairs remained as shown in Figure 1(a) [29].

Weighted correlation network analysis (WGCNA) [27, 28] is widely used for constructing coexpression network based on gene expression data and implementing network analysis [19, 21, 30]. It summarizes clusters of highly correlated genes by defining a continuous network adjacency which is a power of initial one to reduce the low-adjacency gene pairs. WGCNA analyzes the cluster structure and explores the relationships between modules or that between modules and genes. GCN topological characters can be well studied by WGCNA and this great advantage makes WGCNA one of most used GCN construction methods in research. There are many coexpression module detection methods provided by WGCNA for users to choose their own preferred one. Meanwhile, choosing a suitable threshold is required for GCN construction in WGCNA. One limitation of WGCNA is that its GCN construction is undirected. Other prior knowledge is needed if further regulatory analysis based on GCN is designed.

Link-based quantitative methods in DCGL [26, 31] employ a half-thresholding strategy to construct specific GCNs. That is, if at least one of the two coexpression values of a specific link exceeds the threshold, the link in both coexpression networks from two different conditions is kept [26, 31]. In this way, minute variations are ignored by filtering out those noninformative links whose correlation values in both networks are insignificant. GCNs constructed by DCGL are also without directions.

Gaussian graphical model (GGM) is another approach to construct gene coexpression network [32–34]. Based on the assumption that the covariance of gene pairs follows a multivariate Gaussian distribution, partial correlation between gene pairs is calculated as the degree of correlation after the effects of other genes are removed. Unlike the fact that correlations in PCC-based method are calculated by gene pairs themselves, correlations of gene pair in GGM-based methods take into account information of other genes, which makes GGM-based GCN more similar to real biological network.

Some algorithms are proposed with focus on how to infer the structure of gene correlation relationships. Algorithm for the Reconstruction of Accurate Cellular Networks (ARACNE) [35, 36] is a method of GCN construction, which also pays attention to partial network properties of GCN by counting gene triplets. The Context Likelihood of Relatedness (CLR) [37] algorithm calculates the relative correlation based on the empirical correlations over surrounding genes. MRNET is an iterative feature selection algorithm and uses a maximum relevance and minimum redundancy criterion [38].

DECODE (differential coexpression and differential expression) combines the information from both differential coexpression and differential expression to set up the thresholds systematically based on a chi-square maximization [39].

A recent GCN construction algorithm is proposed by Planar Filtered Network Analysis (PFNA) and Multiscale Embedded Gene Coexpression Network Analysis (MEGENA) [40, 41]. According to this algorithm, GCN is constructed on a spherical surface so that links between gene pairs do not cross the others. They have advantages in extracting most relevant information from similarity matrix from complex network system based on topological sphere.

### 2.2. Regulatory Analysis according to Gene Coexpression Network

After the gene coexpression network is constructed based on the transcriptomic data, regulatory information can be extracted by various regulatory analysis methods from the GCN according to research desires as shown in Figure 1(b). The most common way is to use the prior knowledge of TF-target regulatory relationships or miRNA-target regulatory relationships to highlight the specific regulatory subnetwork [25].

Another important method looking for regulatory elements is clustering method. A major goal of coexpression analysis is discovering biologically related modules or gene groups. In WGCNA, hierarchical clustering method is used to identify highly correlated gene subnetworks [27]. Significantly compact subclusters are verified by the average shortest path distance within each cluster over the cluster size. Nonnegative matrix factorization clustering method [42–44] is also employed to clustering coexpressed genes with features of interests.

According to topological structure of gene coexpression network, some interesting characters such as hub genes can help to explain regulatory function contained in the GCN. Connectivity in GCN presents how a gene connects to other genes and hub genes are the ones with very high level of connectivity. Hub genes are normally connections between different gene modules and should be a specific research focus for investigations into cancer-correlated gene modules. For instance, Yang et al. build gene coexpression networks based on transcriptomic and clinical data of four cancer types and discovered that prognostic mRNA genes tended not to be hub genes [24]. They suggested that hubs genes coordinate genes over different pathways to participate in the regulatory processes. Chou et al. investigated endometrial cancers (ECs) hub genes by constructing WGCNA coexpression network and these hub genes are involved in antigen processing, cell adhesion, and cell-cycle regulation [21]. On the other hand, loss of connectivity in coexpression network is a common topological trait among the different kinds of cancer [45].

### 2.3. Differential Regulatory Comparison between Different Conditions

Distinguishing different regulatory elements between different conditions such as tumor and normal tissue, or different cancer types, or even cross-species (human and mouse) [46] help to understand the dysfunctional regulation.

There are two ways to perform differential regulatory comparison between different conditions. The first way is to construct gene coexpression network based on each condition and compare the difference between constructed GCNs to extract different regulation elements as shown in Figure 1(c) [24, 30]. The other way is to calculate the significantly different correlation between various conditions and build a network based on these selected gene pairs [47, 48]. Differential regulatory comparison between different conditions is able to find the differential genes or gene modules across different conditions, providing useful information as well. In DCGL v2, differentially coexpressed TFs are defined as differential regulated genes (DRGs), and DRGs are ranked for prioritizing regulators that are putatively causative to the phenotype of interests in DRrank function in the R package of DCGL2 [25]. For example, RIF algorithm in DRrank function combines three types of transcriptomic information and assigns a high score to those TFs that are “cumulatively most differentially wired to the abundant most differentially expressed genes” [25, 49].

## 3. Applications of Differential Regulatory Analysis Based on GCN in Cancer Research

Recently, differential regulatory analysis based on GCN is applied in more and more cancer studies. In the following, we give some examples of its applications and summarize advantages of this integrative method.

### 3.1. Revealing Dysfunctional Regulatory Genes and Subnetworks in Cancer Research

The direct advantage of differential regulatory analysis (DRA) is that DRA is able to distinguish dysfunctional regulatory subnetworks or pathways in cancer status. For example, Jiang et al. constructed highly preserved gene ontology biological process (GO_BP) gene coexpression network and prostate cancer coexpression network by using WGCNA approach. With regulatory analysis they discovered 548 GO_BP coexpression modules and 294 prostate cancer coexpression modules. By comparing the difference of these modules, they identified 55 conserved prostate cancer coexpression modules [30]. And there are five modules which are significantly enriched with prostate cancer candidate genes. These five modules are featured with regulation of apoptosis, response to stress, cellular localization, and protein localization [30]. Udyavar et al. performed coexpression network construction based on a dataset of combined normal, adenocarcinoma, squamous cell carcinoma, and small-cell lung cancer (SCLC) tissue specimens by WGCNA. They compared the distribution of significant modules across four types of samples and derived an SCLC-specific hub network classifier and identified spleen tyrosine kinase as candidate biomarker and therapeutic target for SCLC [19].

Cancer is considered as a complex disease with multilevel progressing process. DRA based on GCN is able to bring light to dynamic regulatory relationships of cancer in its different progress levels. For instance, Cao et al. first constructed gene coexpression networks of normal, adenoma, and carcinoma-specific gastric carcinogenesis to decrease the searching space for potential regulatory genes [48]. After these potential regulatory genes are acquired, three differential networks are constructed. By comparing constructed differential networking information and signaling pathway information of three developing stages, the regulation roles of GATA6 and ESRRG and their signaling pathways in gastric carcinogenesis were suggested [48]. This work frame is prospective and extendable to other cancer researches. Wu et al. also performed a system-level study of gastric cancer by constructing five phenotype-specific coexpression networks [50]. Their comparison analysis of connectivity reveals that hub genes which only exit in the normal networks play important roles in gastric tumorigenesis and hub genes only related to tumor networks are enriched in specific biological terms. Ruan et al. identified specific pathways associated with renal cell carcinoma (RCC) based on differentially coexpressed links which were detected by three methods: Pearson's correlation, Bayesian network, and WGCNA. These RCC-related pathways help to explain underlying regulatory mechanisms of RCC [29]. Khosravi et al. built independent gene regulatory networks from each prostate cancer and found critical transcription factors involved in prostate cancer based on hub type variation [51]. These dynamic network studies across cancer stages well reveal the change of regulatory patterns during the progression of cancers.

### 3.2. Practical and Beneficial in Multilevel Network Analysis When Integrated with miRNAs and lncRNAs Data

With the emerging roles of microRNAs (miRNAs) and long noncoding RNAs (lncRNAs) in gene regulatory networks, more and more genomic studies on miRNAs and lncRNAs are performed in cancer research aspects [52–58]. By integrating genomic miRNAs and lncRNAs with mRNAs data to construct multi-level co-expression network and analyze differential regulatory mechanism, the understanding of carcinogenesis functions of miRNAs and lncRNAs can be greatly improved from the views of system level.

For example, Lin et al. constructed a cross-cancer miRNA differential coexpression network and identified two potential miRNA-regulated oncomodules associated with poor survival outcomes in patients [59]. This study suggested that disruption of miRNA positive coexpression in cancer might contribute to cancer development. There are many efforts made to discover the regulatory action of lncRNAs in cancer [56, 57]. DRA based on coexpression network is also extendable to lncRNAs-cancer gene network analysis. InCaNet is a data resource which contains precalculated significant coexpression pairs of 9641 lncRNAs and 2544 well-classified cancer genes in 2922 matched TCGA samples [60]. And InCaNet helps to explore regulatory functions of particular lncRNA-cancer gene interaction in cancer studies. Most lncRNAs' regulatory functions are unknown and DRA based on lncRNAs-cancer gene network has the exact ability to perform the predictions. According to the assumption that genes or nodes in a subgroup may execute similar functions, Cogill and Wang identified a list of previously uncharacterized lncRNAs coexpressed with key cancer genes in their study [20] and Hao et al. inferred lncRNAs related to esophageal squamous cell carcinoma ESCC from constructed coexpression network and differential regulatory analysis [61]. Moreover, an integrated miRNA-mRNA-lncRNA coexpression network analysis was performed by Wu et al. to study the oestrogen receptor-regulated transcriptome in breast cancer [62]. All these multilevel studies expand our understanding of regulatory mechanism in cancer biology.

### 3.3. Applicable in Medical and Pharmaceutical Aspects

Since DRA based on GCN is a system-level analysis method and explores the regulatory mechanisms of diseases, it has been widely applied in medical and pharmaceutical aspects. By integrating other pieces of information such as clinical information or drug-target genes information, DRA based on GCN has more potential to contribute to theoretical base of medical and pharmaceutical researches.

For example, prognostic genes are very important for cancer prognosis and treatment. By integrating survival information, Yang et al. studied the system-level prognostic genes across four cancer types by DRA based on GCN [24]. Discovering new biomarkers or molecular subtypes of cancer is also valuable for stratification in clinical studies. DRA-based signature has the ability to classify patients into different subtypes with different clinical results. Meanwhile, DRA-based signature is featured with different regulatory patterns of each subtype. Wu et al. revealed a novel three-transcription-factor signature including AHR, NFIL3, and ZNF423 for glioma molecular subtypes by DRA based on GCN. This three-gene DRA-based signature clusters glioma patients into three major subtypes which are significantly different in patient survival as well as transcriptomic patterns [47]. Jin et al. captured a 12-gene network module of ovarian cancer by constructing weighted survival and differential coexpression network and this module shows a close correlation with cell death [63]. All these prognostic DRA studies based on GCN help to provide a more accurate survival prediction. Moreover, DRA-based prognostic signature has more potential to explore carcinogenesis mechanisms which lead to a better precision medicine in cancer diagnosis and treatment.

## 4. Discussion

Regulatory analysis is always a focal point in biological research. Understanding the function of each regulatory element in biological process is fundamental and challenging. Large-scale and multilevel sequencing data provide more opportunities to reveal molecular regulatory mechanism from the systematic viewpoint. Differential regulatory analysis is designed for distinguishing the differential regulatory elements in different conditions or dysfunctional regulation specific for an abnormal condition. For example, cancer is considered as a complex genetic disease and different phonotypes in cancer embody regulatory level mechanisms. Dysfunctional regulatory elements take priority in carcinogenesis studies because of their important roles in regulation as well as their potential in cancer treatments.

In the early past decade, gene expression data for specific phenotype is very limited and researchers had to use various cell lines data to construct conceptual gene regulatory networks [14]. Then it is very difficult to explain the differential regulatory relationships between tumor types. Recently, with the accumulation of large-scale and multiscale data, researchers are able to apply differential regulatory analysis to identify specific regulatory patterns in a given cancer type. Since differential regulatory analysis based on coexpression network has a system-level property, it has great strength to discover underlying molecular mechanism and dysfunctional regulatory elements from large-scale data of complex system. With the development of differential regulatory analysis based on coexpression network itself and its applications in more and more genomic and big data research, it presents its essential and prospective role in cancer research.

Technically, construction of coexpression networks as the basic starting point of differential regulatory analysis plays important and critical role in the whole investigation process. Therefore, both the data sets and the way to construct networks must be carefully examined. For example, Ballouz et al. suggest minimal experimental criteria to obtain useful functional connectivity and topology information of coexpression with microarrays greater than 20 samples and read depth greater than 10 M per sample [64]. Selection of differential regulatory genes relies on the method or algorithm chosen in DRA based on GCN [22, 65, 66]. For instance, differential analysis between two conditions, which is followed by regulatory analysis, can be performed after two gene coexpression networks are constructed. Sometimes, regulatory analysis based on coexpression networks between two conditions is conducted first and comparison of differential regulatory elements is performed later. All these steps in current differential regulatory analysis methods are relatively flexible depending on the aim of specific research and information available. The DRA based on GCN method might need further standardization and refinement to better serve more carcinogenesis research in medical and pharmaceutical fields.

## 5. Conclusion

In this review, we summarize the paradigm of differential regulatory analysis based on coexpression network of transcriptomic data and the applications of differential regulatory analysis based on GCN in cancer research. Differential regulatory analysis based on GCN is demonstrated as a necessary and potential tool to reveal underlying molecular mechanism in basic functional genomic research as well as practical carcinogenesis studies.
